# Mechanisms of asymmetry in sea surface temperature anomalies associated with the Indian Ocean Dipole revealed by closed heat budget

**DOI:** 10.1038/s41598-021-01619-2

**Published:** 2021-11-25

**Authors:** Mai Nakazato, Shoichiro Kido, Tomoki Tozuka

**Affiliations:** 1 Department of Earth and Planetary Science, Graduate School of Science, The University of Tokyo, 7-3-1 Hongo, Bunkyo-ku, Tokyo, 113-0033 Japan; 2 Application Laboratory (APL), Research Institute for Value‐Added‐Information Generation (VAiG), Japan Agency for Marine‐Earth Science and Technology (JAMSTEC), Yokohama, Japan

**Keywords:** Physical oceanography, Climate sciences, Ocean sciences

## Abstract

The Indian Ocean Dipole (IOD) is an interannual climate mode of the tropical Indian Ocean. Although it is known that negative sea surface temperature (SST) anomalies in the eastern pole during the positive IOD are stronger than positive SST anomalies during the negative IOD, no consensus has been reached on the relative importance of various mechanisms that contribute to this asymmetry. Based on a closed mixed layer heat budget analysis using a regional ocean model, here we show for the first time that the vertical mixing plays an important role in causing such asymmetry in SST anomalies in addition to the contributions from the nonlinear advection and the thermocline feedback proposed by previous studies. A decomposition of the vertical mixing term indicates that nonlinearity in the anomalous vertical temperature gradient associated with subsurface temperature anomalies and anomalous vertical mixing coefficients is the main driver of such asymmetry. Such variations in subsurface temperature are induced by the anomalous southeasterly trade winds along the Indonesian coast that modulate the thermocline depth through coastal upwelling/downwelling. Thus, the thermocline feedback contributes to the SST asymmetry not through the vertical advection as previously suggested, but via the vertical mixing.

## Introduction

The Indian Ocean Dipole (IOD)^1^ is a dominant interannual climate mode of the tropical Indian Ocean that develop through the Bjerknes feedback^2^. During positive IOD (pIOD) events, negative sea surface temperature (SST) anomalies appear in the southeastern tropical Indian Ocean and positive SST anomalies develop in the western tropical Indian Ocean. Such SST anomalies first emerge during boreal summer and reach their peak in autumn, followed by rapid decay in winter. Anomalies with the opposite signs appear during negative IOD (nIOD) events. Since the IOD is known to affect not only coastal countries of the Indian Ocean^3–7^, but also remote regions^8–10^, better understanding and more accurate forecasts of the IOD are of particular importance.

One of the essential characteristics of the IOD is its asymmetry; negative SST anomalies in the eastern pole during the pIOD are stronger than positive SST anomalies during the nIOD^11^. Due to such asymmetry in SST anomalies, the distribution and amplitude of the atmospheric teleconnection are asymmetric^12^. As a result, impacts on precipitation^13,14^ and marine ecosystems^15^ are also not symmetric between pIOD and nIOD. Thus, understanding of the mechanisms responsible for the asymmetry is crucial.

In this regard, the first study on the cause of asymmetry in SST anomalies over the eastern pole of the IOD emphasized the importance of the nonlinear zonal and vertical advection based on a mixed layer heat budget analysis^11^. More specifically, they pointed out that the nonlinear zonal advection term leads to anomalous cooling during both pIOD and nIOD; negative zonal SST gradient anomalies are advected by westward current anomalies during the pIOD, while positive zonal SST gradient anomalies are advected by eastward current anomalies during the nIOD. Similarly, both anomalous upward advection of anomalous positive vertical temperature gradient during the pIOD and anomalous downward advection of anomalous negative vertical temperature gradient during the nIOD contribute to the SST cooling. Thus, such nonlinear dynamical heating anomalies act to amplify SST anomalies associated with the pIOD and damp those associated with the nIOD. Also, they argued that during the pIOD, the negative SST-cloud-shortwave radiation feedback is weaker and acts to enhance the anomalous SST cooling, because the SST becomes cooler than the convective threshold and an anomalous increase in shortwave radiation is bounded.

Although the importance of the SST-cloud-shortwave radiation feedback is further highlighted^12^, this was challenged by subsequent studies^16–18^, which showed that the feedback actually weakens the asymmetry. It was pointed out that such discrepancies are due to SST biases in the ocean assimilation product and spurious trends in precipitation seen in the atmospheric reanalysis data used by previous studies^16^.

Also, contrasting results were obtained regarding the role of the thermocline feedback^12,16–19^. It was proposed that the SST in the eastern pole is more sensitive to an anomalous shoaling of the thermocline than to an anomalous deepening, because the mean thermocline there is relatively deep^19^. Their hypothesis was verified by a modeling study^18^: By conducting sensitivity experiments with an ocean general circulation model (OGCM), it was shown that the negative SST skewness in the eastern pole is reproduced even under sinusoidal zonal wind stress anomalies. Also, the importance of the thermocline feedback was indicated using various observational data^17^. On the other hand, a study that conducted a mixed layer heat budget analysis taking mixed layer depth (MLD) variations into account demonstrated that the contribution from the thermocline feedback, which was quantified by the vertical advection, is small^12^. However, the validity of this result was questioned owing to large temperature biases in the assimilation product used in that study^16^.

Although various mechanisms have been proposed for the amplitude asymmetry in the IOD, there are some discrepancies among the past studies and no consensus regarding the relative importance of individual processes. One reason for such discrepancies may be the use of an offline mixed layer heat budget analyses; their budget was not closed and accompanied large residuals arising from uncertainties in estimations of each term. Indeed, several recent studies delineated the complexity in driving mechanisms of SST anomalies associated with the pIOD by conducting online heat budget analyses using OGCMs^20–22^. Although we need to pay attention to model biases, such closed heat budget analyses can help us identify the key mechanisms and may provide a solution. Also, despite the fact that the MLD undergoes large variations at different timescales, past studies except for Hong and Li^12^ assumed constant MLD when conducting a heat budget analysis. Motivated by the above, we attempt to reveal the mechanisms of the asymmetry in SST anomalies over the eastern pole of the IOD by conducting an online mixed layer heat budget analysis using a regional ocean model that allows exact closure. To examine the relative role of oceanic and atmospheric nonlinearity, heat budget terms with and without normalization by the atmospheric forcing are compared.

## Results

### Mixed layer heat budget analysis of the eastern pole

**Figure 1:**
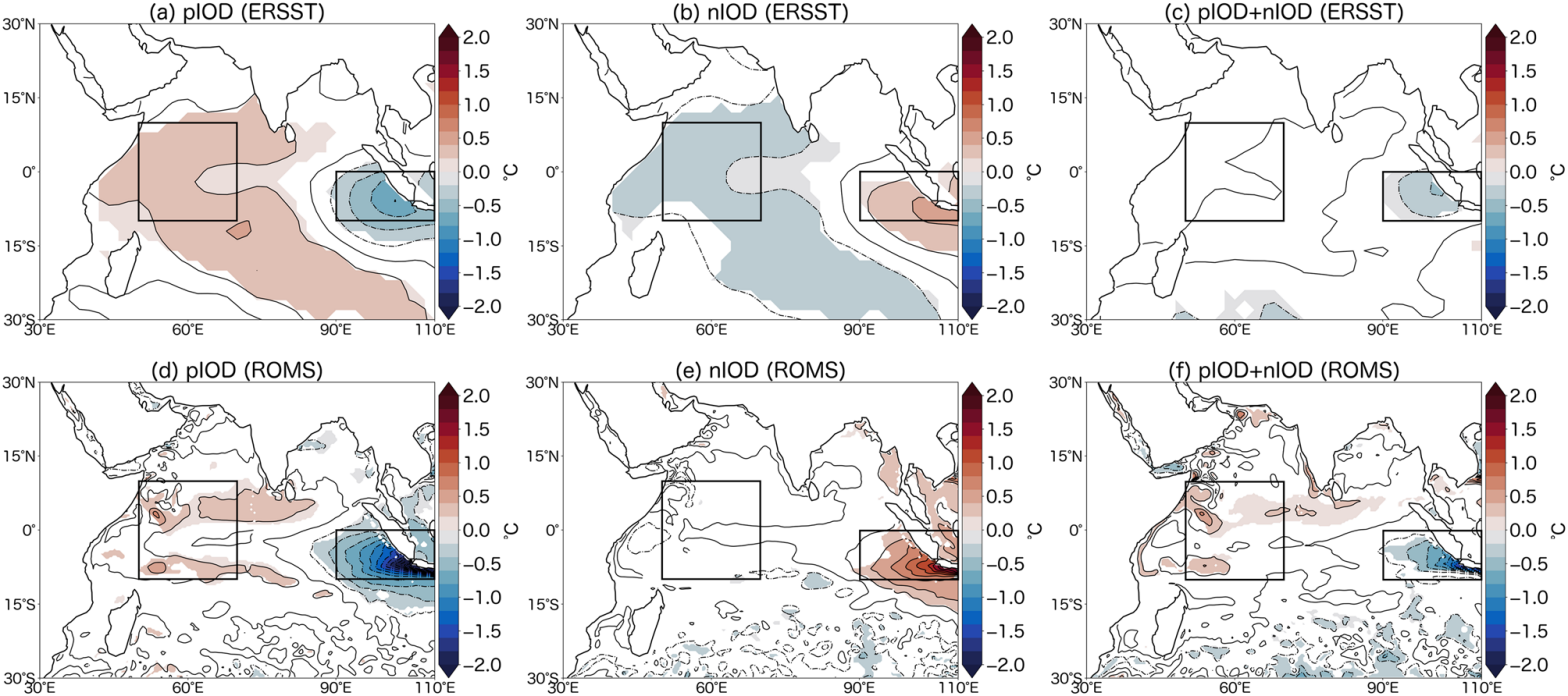
Sea surface temperature (SST) anomalies associated with the positive Indian Ocean Dipole (pIOD) and negative Indian Ocean Dipole (nIOD). Composites of SST anomalies (in °C) during September (0) to November (0) of pIOD years (left) and nIOD years (middle) from the ERSST (**a**–**c**) and the ROMS (**d**–**f**). Their sums (i.e., pIOD plus nIOD) are also presented (right). The contour intervals are 0.2 °C. Anomalies significant at the 95% confidence level by a two-tailed t test are shaded. The boxes represent the western and eastern poles of the Indian Ocean Dipole (IOD). Although an asymmetric component is often expressed in terms of (pIOD + nIOD)/2, figures in this paper use pIOD + nIOD to ease comparison with past studies on the SST asymmetry of the IOD.

Figure 1 shows composites of observed and modeled SST anomalies for the pIOD and nIOD years (Fig. S1; see “Methods” for how event years are defined). Although the simulated SST anomalies over the eastern pole is overestimated and extend too far to the west and those over the western pole is underestimated compared to the observation especially in the pIOD, the zonal dipole structure and asymmetric amplitude of SST anomalies associated with the IOD are well reproduced. The skewness of SST anomalies is also calculated (see “Methods”) for both model and observation (Fig. S2). Although the skewness is exaggerated in the model, the negative skewness over the eastern pole of the IOD are found in both model and observation.

**Figure 2:**
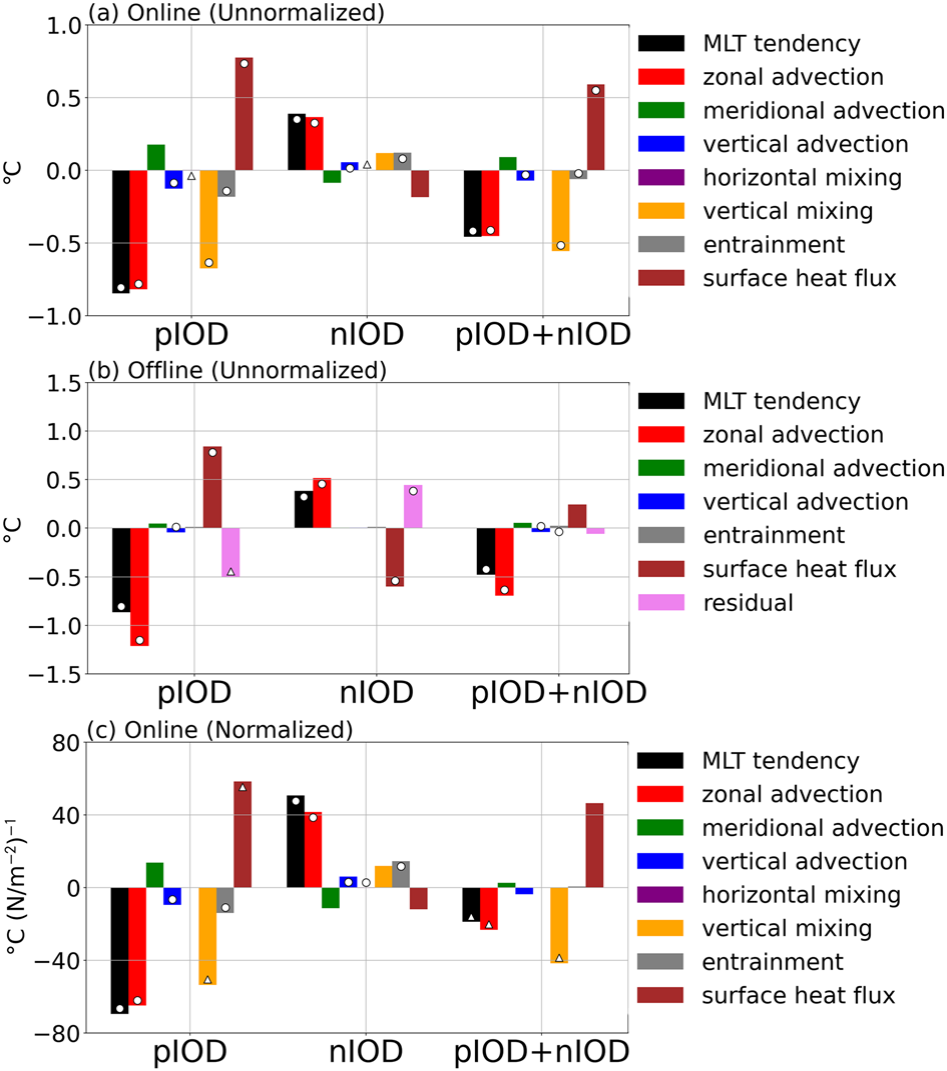
Mixed layer heat budget of pIOD and nIOD years. (**a**) Mixed layer temperature (MLT) tendency (black), zonal advection (red), meridional advection (green), vertical advection (blue), horizontal mixing (purple), vertical mixing (orange), entrainment (grey), and surface heat flux (brown) term anomalies (in °C) integrated over the development phase from April (0) to September (0) for the pIOD (left) and the nIOD (middle). Their sums are also presented (right). The circles (triangles) indicate that anomalies are significant at the 95% (90%) confidence level by a two-tailed t test. (**b**) As in (**a**), but with an offline calculation. The residual term is indicated by a violet bar. (**c**) As in (**a**), but normalized by the atmospheric forcing (i.e., EQUINOO, see “Methods”).

To quantitatively understand the mechanisms of the asymmetric SST variations in the southeastern tropical Indian Ocean, we conduct the mixed layer heat budget analysis (see “Methods”). Composites of each term of the mixed layer heat budget equation (see Eq. (1) in “Methods”) averaged over the eastern pole of the IOD during the development phase from April (0) to September (0) are shown in Fig. 2a (see Fig. S3 for their time evolution). The anomalous cooling during the pIOD is mostly associated with zonal advection and vertical mixing anomalies and vertical advection and entrainment anomalies make a smaller contribution, while the surface heat flux term tends to damp negative SST anomalies. The importance of zonal advection and vertical terms is consistent with past studies^11,12,20^, but the dominance of the vertical mixing term within three vertical terms differs with past studies^18^. For the nIOD, the zonal advection term contributes dominantly to the anomalous warming, and vertical advection and entrainment anomalies make a minor contribution.

In terms of the asymmetry, the zonal advection and vertical mixing terms strengthen the asymmetry, while the surface heat flux term weakens the asymmetry. Interestingly, the vertical mixing term plays an important role in generating the asymmetry, in contrast to past studies that underscored the importance of the zonal advection term.

Such discrepancy may be caused by the difference between online and offline calculations of the mixed layer heat budget^20,22^. Indeed, when we perform an offline heat budget analysis using monthly-averaged outputs from the model, the asymmetry in the zonal advection term is exaggerated, while the residual term, which implicitly represents the vertical mixing term, does not exhibit a significant asymmetry (Fig. 2b). These results imply that an offline heat budget analysis may overemphasize the role of zonal advection, as it cannot fully resolve sub-monthly variability, which substantially rectifies onto the mean state and low-frequency variability^23,24^. Thus, caution is required when interpreting results from an offline heat budget analysis.

If the above SST asymmetry were driven by a much larger response of the atmosphere to the SST anomalies during the pIOD, even a linear ocean would display a larger response during the pIOD. To exclude this possibility and check whether the asymmetry in individual terms of the heat budget analysis merely reflects a passive oceanic response to nonlinearity of the atmosphere, we have repeated the mixed layer heat budget analysis by normalizing all terms by EQUINOO of each event (see “Methods”). Figure 2c indicates that even after the normalization, the amplitude of anomalous cooling during the pIOD is greater than that of anomalous warming during the nIOD and both vertical mixing and zonal advection terms contribute significantly to this amplitude asymmetry, with a larger contribution from the vertical mixing term. This is because the amplitude of the anomalous cooling during the pIOD (− 0.85 °C) is about 2.2 times larger than that of the anomalous warming during the nIOD (− 0.39 °C), but the amplitude of easterly wind stress anomalies during the pIOD (1.46 × 10^–2^ N m^−2^) is only about 1.6 times larger than that of westerly wind stress anomalies during the nIOD (9.06 × 10^–3^ N m^−2^). This suggests that the oceanic response is not completely linear and that the IOD asymmetry is not solely a consequence of atmospheric nonlinearities.

**Figure 3:**
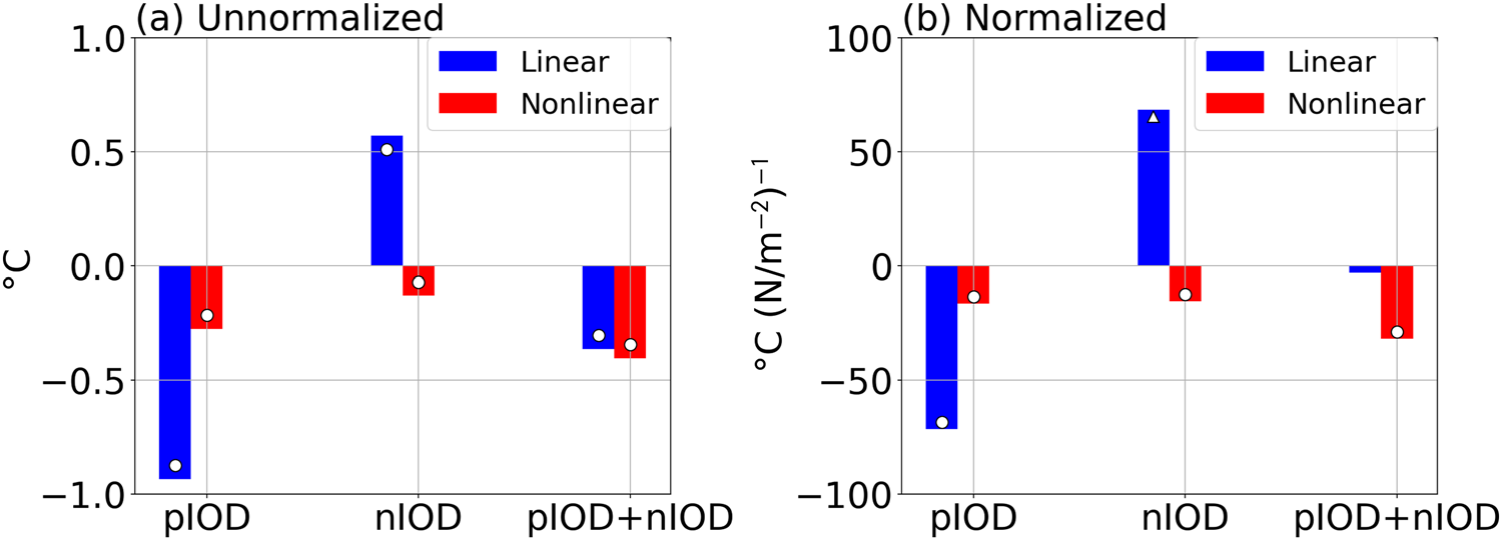
Decomposition of the horizontal advection term. (**a**) Linear (blue) and nonlinear (red) horizontal advection terms (in °C) integrated over the development phase for the pIOD (left) and the nIOD (middle). Their sums are also presented (right). (**b**) As in (**a**), but normalized by EQUINOO. The circles (triangles) indicate anomalies significant at the 95% (90%) confidence level by a two-tailed t test.

A decomposition of the horizontal advection term into linear and nonlinear components in an offline calculation (Fig. 3a) reveals that both components contribute to the asymmetry. Specifically, the linear advection term contributes to the growth of both pIOD and nIOD, but its amplitude is stronger in the pIOD. However, the asymmetry in the linear advection term may be due to that in the strength of the atmospheric forcing; when normalized by EQUINOO, the asymmetry in the linear advection term is weak and not statistically significant (Fig. 3b). On the other hand, the nonlinear advection term contributes to the anomalous cooling during both pIOD and nIOD even after the normalization (Fig. 3b). As a result, it enhances the pIOD, but damps the nIOD. This supports Hong et al.^11^, who suggested that the nonlinear horizontal advection contributes to the asymmetry.

Also, the result that the surface heat flux term weakens the asymmetry supports the conclusions of Cai et al.^16^ and Ogata et al.^18^, but we note that this damping is not statistically significant if we normalize the term with EQUINOO (Fig. 2c). In addition, the vertical advection term enhances the asymmetry, but the amplitude is relatively small. This is in agreement with Hong and Li^12^, but somewhat different from Ogata et al.^18^. Such quantitative differences may be partly due to those in definitions of MLD in the heat budget analysis; Hong and Li^12^ and ours employ variable MLD, whereas Ogata et al.^18^ assumed a constant MLD of 50 m.

### Asymmetry in the vertical mixing term

**Figure 4:**
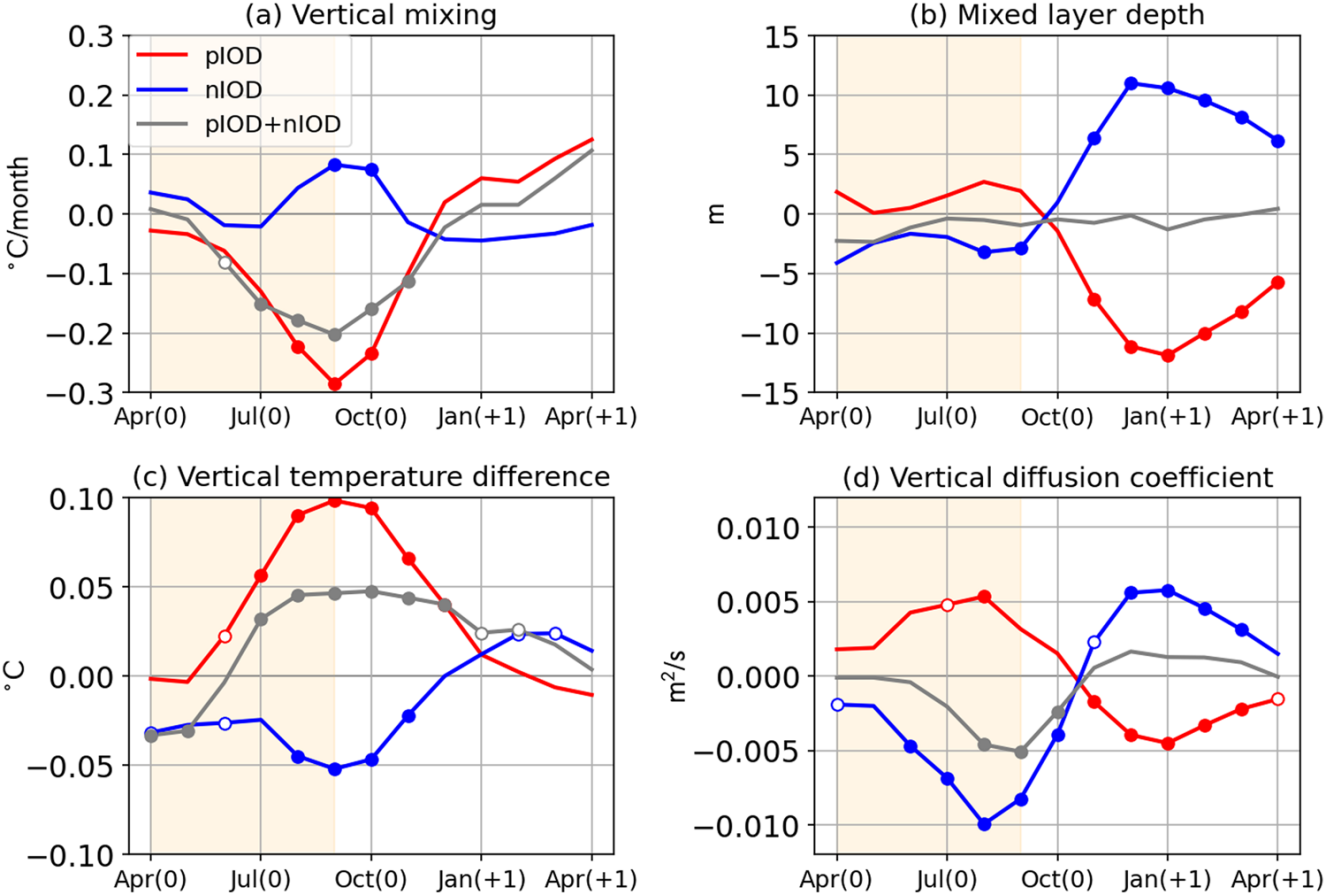
Vertical mixing term and its constituents during pIOD and nIOD years. Time series of composited (**a**) vertical mixing term (in °C/month), (**b**) mixed layer depth (MLD) (in m), (**c**) difference in temperature between the mixed layer and just below it (in °C), and (**d**) vertical diffusion coefficient (in ${\text{m}}^{2}\text{/}{\text{s}}$) anomalies for the pIOD (red), the nIOD (blue), and the sum of pIOD and nIOD events (grey). The closed (open) circles indicate anomalies significant at the 95% (90%) confidence level by a two-tailed t test. The development phase is shaded.

We now focus on the vertical mixing term whose contribution to the amplitude asymmetry of the IOD has not been discussed by previous studies. Since it is not straightforward to clearly distinguish between the effects of vertical mixing and advection at the bottom of the mixed layer in an ocean model that adopts subgrid-scale vertical mixing parameterization, the vertical advection and mixing terms are often grouped as “vertical processes” in many previous studies^12,18^. However, the online analysis used in this study allows us to explicitly separate individual contributions. We first compare the time series of anomalies in MLD, vertical diffusion coefficient, and vertical temperature gradient that constitute the vertical mixing term (Fig. 4, see “Methods”).

**Figure 5:**
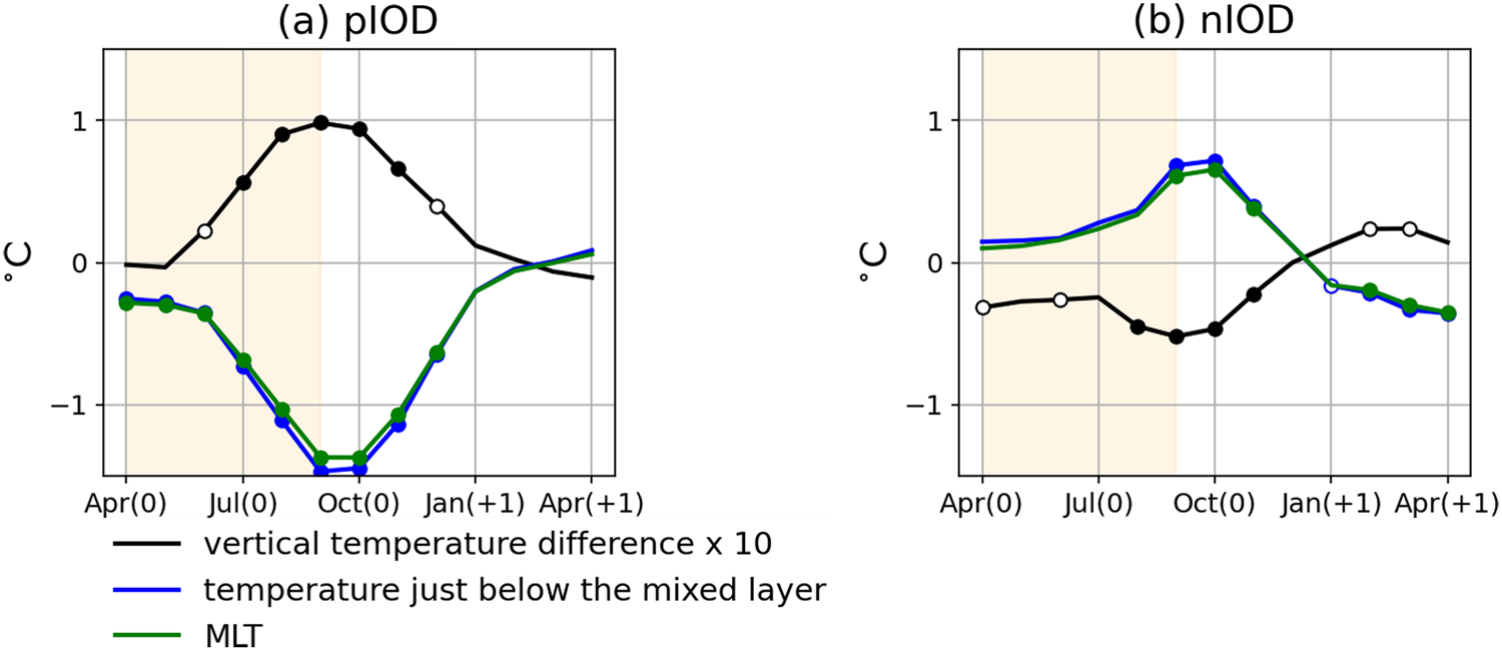
Vertical temperature difference during pIOD and nIOD years. As in Fig. 4, but for vertical temperature difference multiplied by 10 (black), temperature just below the mixed layer (blue), and MLT (green) anomalies (in °C) for (**a**) the pIOD and (**b**) the nIOD.

The vertical temperature gradient, which is calculated by taking the difference in temperature between the mixed layer and just below it, is anomalously large in the pIOD and small in nIOD years (Fig. 4c). However, the amplitude of anomalies is nearly two times larger in the pIOD compared to the nIOD. The anomalous vertical temperature gradient is predominantly attributed to subsurface temperature anomalies caused by thermocline depth variations, whose magnitude is larger than that of the concomitant MLT anomalies for both pIOD and nIOD (Fig. 5).

**Figure 6:**
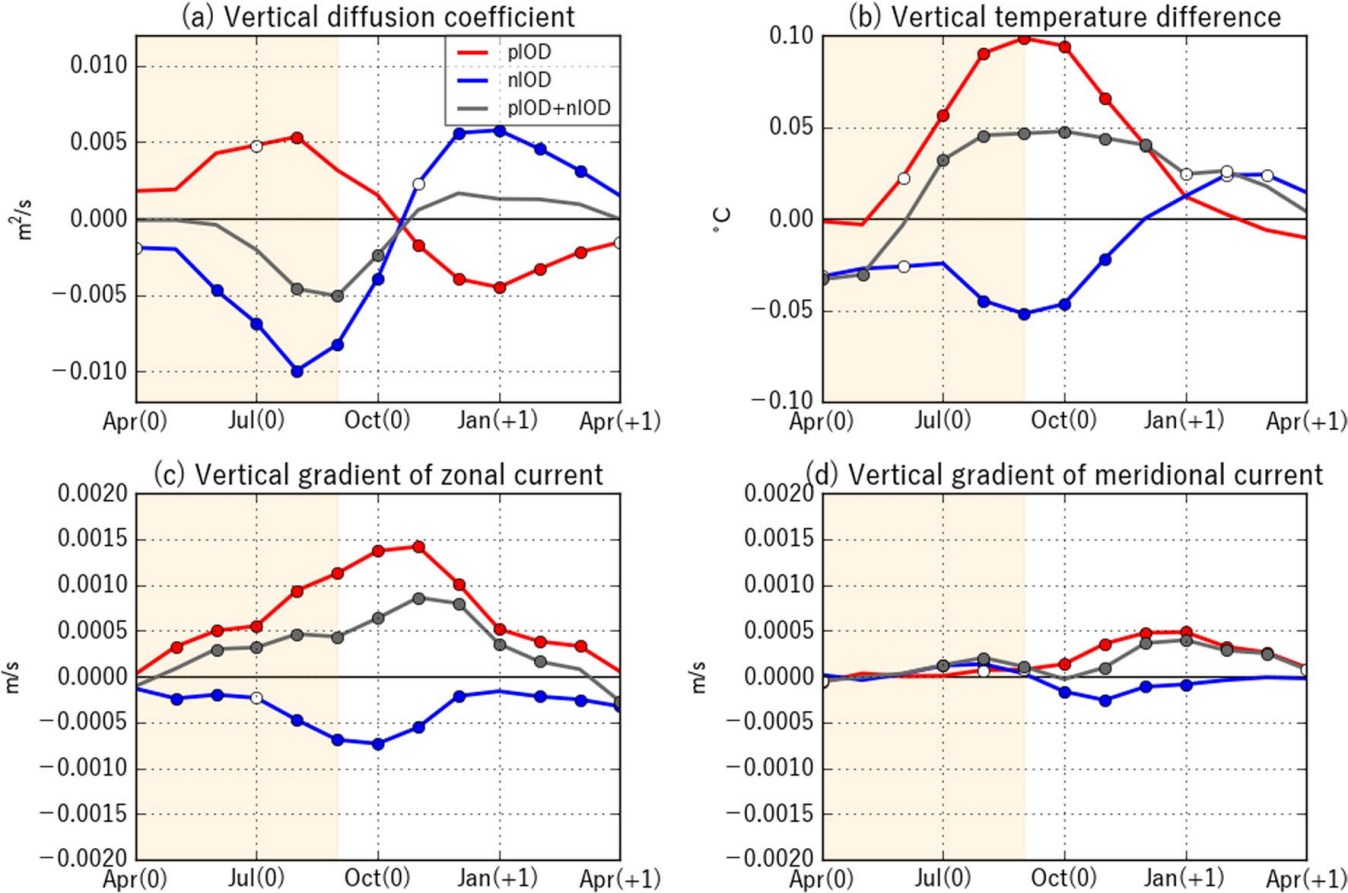
Vertical diffusion coefficients during pIOD and nIOD years. As in Fig. 4, but for (**a**) vertical diffusion coefficient (in m^2^/s), (**b**) vertical temperature difference (in °C), and vertical gradient of (**c**) zonal and (**d**) meridional current (in m/s) anomalies.

Vertical diffusion coefficient anomalies, which measure the strength of the vertical mixing, are positive in the pIOD, but they are smaller in amplitude compared with negative anomalies in the nIOD (Fig. 4d). Considering that vertical diffusion coefficients increase with decreasing stratification and increasing vertical shear, we analyze the vertical temperature gradient and the vertical shear in horizontal currents to gain further insight (Fig. 6). During the pIOD, the vertical shear is strengthened by the intensified southeasterly trade winds and contributes to stronger vertical mixing, whereas the relaxation of the trade winds associated with the nIOD reduces the vertical shear and thus weakens the vertical mixing. On the other hand, the density stratification strengthens (weakens) in the pIOD (nIOD) reflecting the subsurface temperature anomalies (Figs. 4c, 5). Thus, the anomalous stratification acts to counteract vertical diffusion coefficient anomalies associated with vertical shear anomalies in both phases. We note that salinity anomalies also contribute to the changes in density stratification, but their relative contribution to the density stratification is smaller than that of temperature anomalies^25^. Since the magnitude of positive anomalies in the vertical diffusion coefficient in the pIOD is smaller than that of the negative anomalies in the nIOD, the above results suggest that the contribution of the stratification and the vertical shear cancel each other out more strongly in the pIOD, although more detailed analyses are required for quantitative discussions.

The mixed layer is anomalously shallow during the nIOD, but no statistically significant MLD anomalies are found during the pIOD (Fig. 4b). Weaker anomalies during the pIOD can be explained as follows; anomalous deepening of the mixed layer due to the stronger vertical shear (Fig. 6c,d) and/or wind-induced mixing are counteracted by strong positive surface heat flux anomalies (Fig. 2) and enhanced density stratification (Fig. 6b). Similar compensation between dynamical and thermodynamical forcing also occurs for the nIOD, leaving weaker MLD anomalies (~ 2 m).

**Figure 7:**
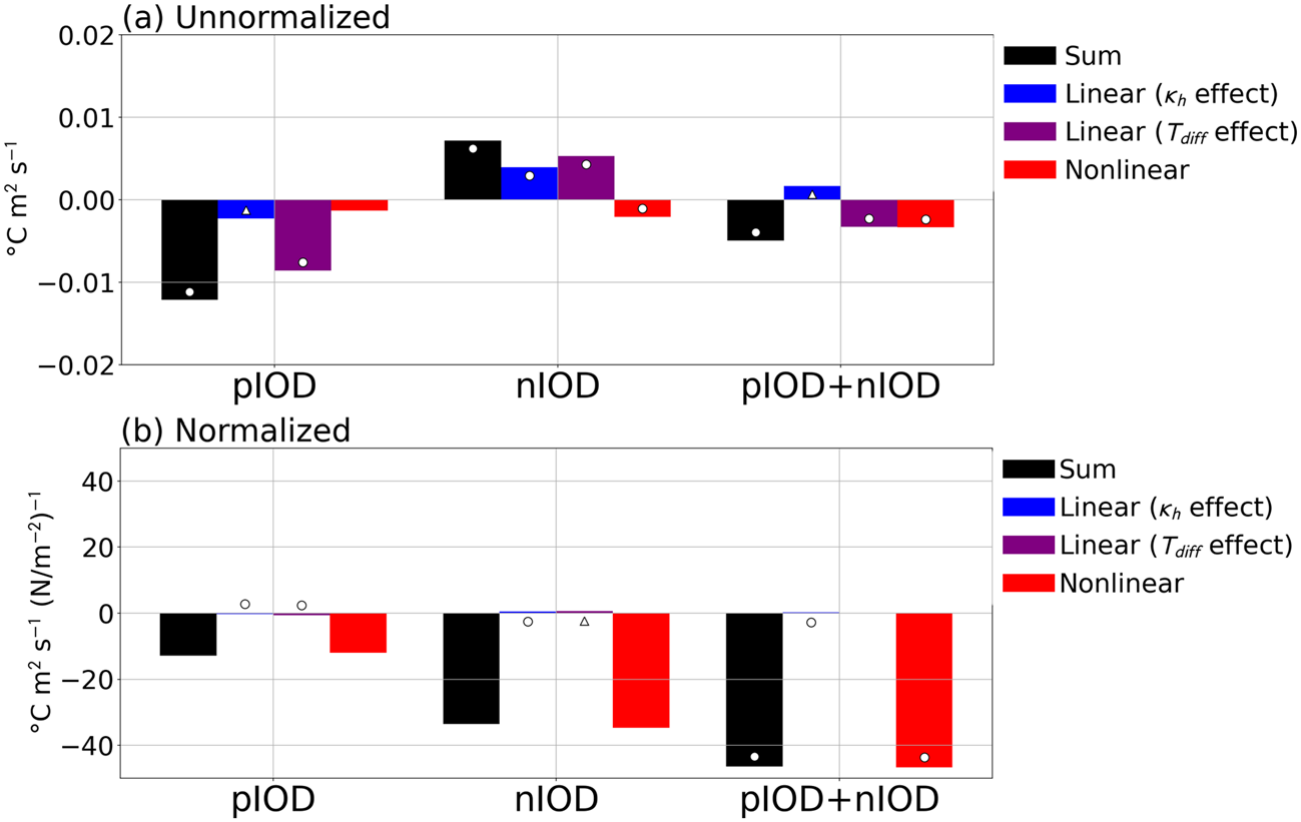
Decomposition of the vertical mixing term. (**a**) Linear terms associated with the vertical diffusion coefficient anomaly (blue; the first term on the right hand side) and the temperature difference anomaly (purple; the second term on the right hand side), nonlinear term (red; the third term on the right hand side), and their sum (black; the left hand side term) in Eq. (3) integrated over the development phase for the pIOD (left) and the nIOD (middle) (in °C m^2^ s^−1^). Their sums are also presented (right). (**b**) As in (**a**), but normalized by EQUINOO. The circles (triangles) indicate anomalies significant at the 95% (90%) confidence level by a two-tailed t test.

A decomposition of the vertical mixing term into linear and nonlinear components is also performed in an offline calculation (Fig. 7a; see “Methods”). For both pIOD and nIOD, the linear term associated with the temperature difference anomaly [i.e., the second term on the right hand side of Eq. (3)] contributes dominantly to the anomalous SST cooling and warming, respectively, and the linear term associated with the vertical diffusion coefficient anomaly [i.e. the first term on the right hand side of Eq. (3)] also makes substantial contribution during the nIOD. Regarding the asymmetry, the linear term associated with the temperature difference anomaly and the nonlinear term (i.e., the second and third terms on the right hand side of Eq. (3), respectively) make a comparable contribution. However, the asymmetry in the vertical mixing term is solely due to that in the nonlinear term when normalized by EQUINOO (Fig. 7b). The physical processes represented in the nonlinear term can be explained as follows: During the pIOD, positive temperature difference and vertical diffusion coefficient anomalies lead to enhanced cooling; i.e., enhanced mixing of anomalously cold water takes place. On the other hand, negative temperature difference and vertical diffusion coefficient anomalies during the nIOD contribute to anomalous cooling. In other words, a weakening of the vertical mixing during the nIOD reduces incorporations of anomalously warm subsurface water to the surface, leading to suppression of further SST warming. Thus, the thermocline feedback is suggested to contribute to the asymmetry mainly through the vertical mixing. This is in contrast to Ogata et al.^18^, who suggested the importance of the thermocline feedback through vertical advection.

## Conclusions and discussions

In this study, we have investigated the asymmetry in SST anomalies over the eastern pole of the IOD for the first time based on a completely closed mixed layer heat budget analysis using a regional ocean model that realistically reproduces the main features the IOD. In addition to the contributions from the nonlinear advection and the thermocline feedback proposed by previous studies^11,12,18^, we have found for the first time that the vertical mixing term makes an important contribution to the SST asymmetry. This is true even when its contribution is normalized by the atmospheric forcing, indicating the importance of oceanic nonlinearity in the generation of the SST asymmetry. A further decomposition of the vertical mixing term indicates that nonlinearity in the anomalous vertical temperature gradient associated with subsurface temperature anomalies and anomalous vertical mixing coefficients strengthens the SST asymmetry.

**Figure 8:**
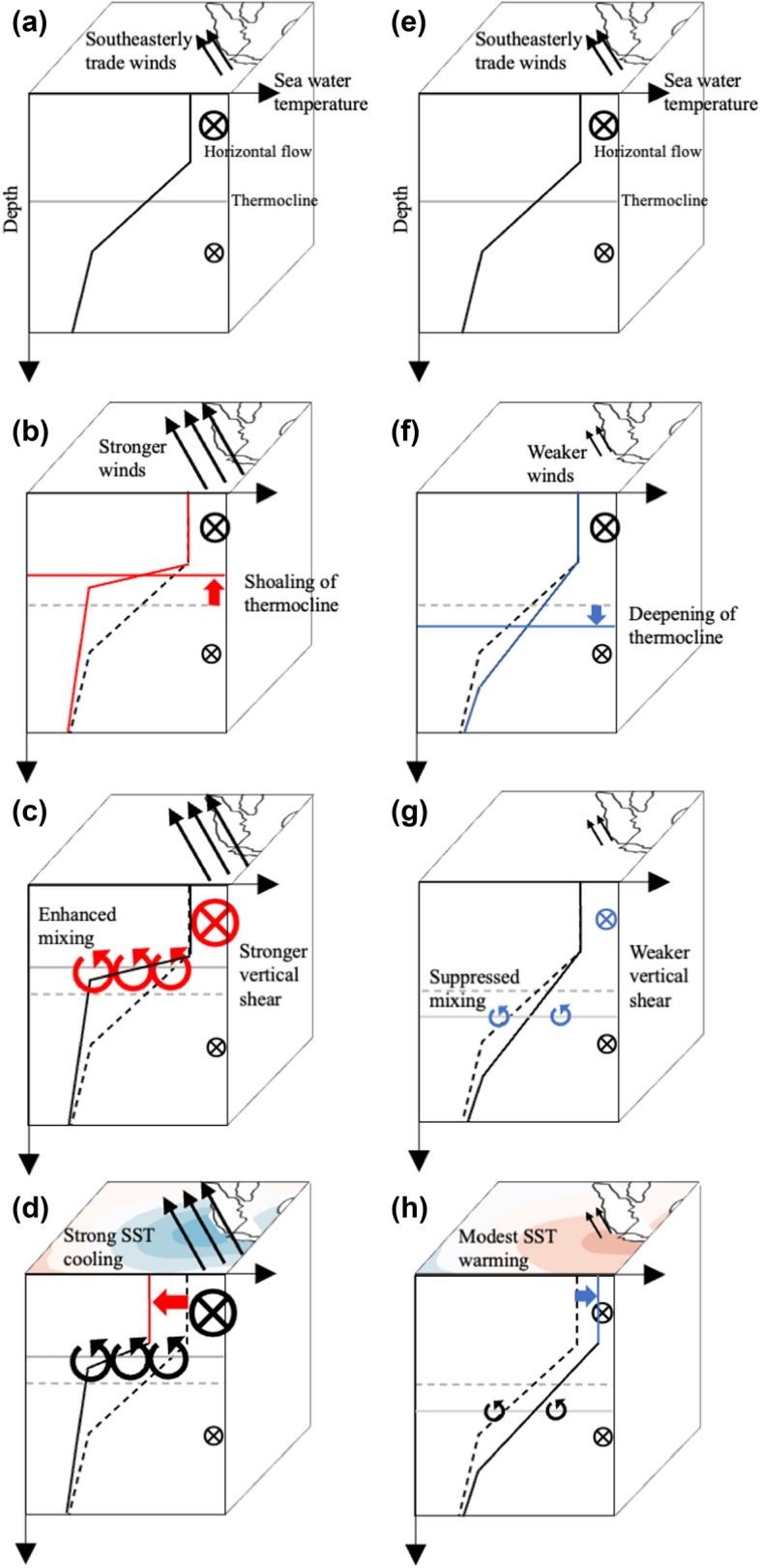
Schematic diagrams explaining how the vertical mixing contributes to the SST asymmetry. Schematic representation of the anomalous vertical mixing term for the pIOD (**a**–**d**) and the nIOD (**e**–**h**). The shading in (**d**) and (**h**) indicates SST anomalies.

Figure 8 schematically explains how the vertical mixing contributes to the SST asymmetry. During the pIOD (Fig. 8a–d), the anomalously strong southeasterly trade winds along the Indonesian coast enhance coastal upwelling and lead to the shallower thermocline and negative temperature anomalies just below the mixed layer (Fig. 8b). Moreover, due to the stronger southeasterly wind anomalies, the surface northwestward currents are enhanced and the vertical shear is strengthened (Fig. 8c). Although the anomalous cooling just below the mixed layer enhances the stratification and favors a smaller vertical diffusion coefficient, this effect seems to be overwhelmed by the vertical shear effect and the vertical diffusion coefficient becomes anomalously positive. Thus, the negative SST anomalies are generated by more effective mixing of anomalously cold water from below (Fig. 8d). The opposite occurs during the nIOD (Fig. 8e–h) and less effective mixing of anomalously warm subsurface water damps the anomalous warming associated with the nIOD. To assess the validity of our finding, however, more coordinated modelling frameworks that specifically focus on the vertical mixing (e.g., a one-dimensional modelling framework^26^) as well as direct observations of microstructure turbulence are required.

To take account of the difference in the strength of atmospheric forcing between pIOD and nIOD and to quantify the relative importance of nonlinearity in the oceanic processes, we have normalized heat budget terms with EQUINOO in the present study. However, there may be some room for discussions regarding how we define the atmospheric forcing. Moreover, this normalization assumes that the oceanic response are completely linear and that the IOD asymmetry is a sole consequence of atmospheric nonlinearities. In reality, however, stronger wind anomalies during the pIOD are at least partly due to a linear response to larger SST anomalies generated by oceanic processes. For instance, the amplitude of easterly wind stress anomalies (i.e. EQUINOO) during the pIOD (1.46 × 10^–2^ N m^−2^) is about 1.6 times larger than that of westerly wind stress anomalies during the nIOD (9.06 × 10^–3^ N m^−2^), but the amplitude of surface westward current anomalies during the pIOD over the same region (1.20 × 10^–1^ m s^−1^) is about 2.0 times larger than that of surface eastward current anomalies during the nIOD (5.99 × 10^–2^ m s^−1^). This may be because wind stress anomalies are trapped more efficiently near the surface during the pIOD owing to anomalously shallow thermocline over the central-to-eastern equatorial Indian Ocean, and stronger equatorial zonal current anomalies are induced near the surface even if wind stress anomalies with the same amplitude are applied. Importance of a nonlinear oceanic response to the atmospheric forcing has also been suggested for the El Niño/Southern Oscillation (ENSO)^27^; it is indicated that wind stress anomalies associated with El Niño are more efficiently trapped in the upper ocean of the western tropical Pacific owing to shallower thermocline and contribute to more rapid decay of El Niño than La Niña^28^. Hence, a separate study conducting a hierarchy of model experiments with symmetric wind forcing^18,29^ may be useful to further investigate the IOD asymmetry.

Although the main focus of this study is how oceanic nonlinearity contributes to the IOD asymmetry, our analyses with the normalization by the EQUINOO suggest that atmospheric nonlinearity also plays a role. Since the atmospheric nonlinearity has been shown to play an important role in generating the asymmetry in ENSO^27^, it will be illuminating to conduct an atmospheric model experiment in which symmetric SST anomalies associated with the positive and negative IOD are imposed.

It is projected that the IOD becomes less asymmetric under global warming^19^, but coupled models suffer from biases in reproducing the skewness of the IOD^30–32^. Since the skewness is one of the essential characteristics of the IOD, it is necessary to figure out the root cause of this model bias for a more reliable future projection. This study suggests that such bias may partly be alleviated by more realistically simulating the physical processes summarized in Fig. 8.

Asymmetry in SST anomalies is not limited to the IOD. For instance, the ENSO shows strong asymmetry with stronger El Niño and weaker La Niña^27,33^. Various processes, such as the nonlinear advection, tropical instability waves, state-dependent stochastic forcing, nonlinear oceanic wave response, bio-physical feedback, and atmospheric nonlinearities have been proposed as the origin of the asymmetry in the ENSO^27,34,35^, but their relative importance is still under debate as in the IOD. To gain quantitative insight into the mechanisms of the amplitude asymmetry, an online mixed layer heat budget analysis that allows an exact closure is essential, as offline analysis introduce large uncertainties in estimations of contributions from each process. Applications of the present framework to other climate modes will be an interesting topic for future research.

## Methods

### Observational data

We use SST data from the Extended Reconstructed Sea Surface Temperature version 3 (ERSST v3)^36^ from 1950 to 2017 with a horizontal resolution of 2°. To focus on interannual variations, we have applied 3-month running mean to filter out high frequency variabilities and then removed decadal and longer variations with 73-month running mean from all observational data and model outputs prior to our analyses.

### Positive and negative IOD years

We define pIOD and nIOD years based on the Dipole Mode Index (DMI) averaged from September to November (Fig. S1; solid). The DMI is defined as the difference between the area-averaged SST anomalies in the western pole (50°E–70°E, 10°S–10°N) and the eastern pole (90°E–110°E, 10°S–Equator)^1^. Following Ummenhofer et al.^37^, who took into account the asymmetric nature of the IOD, years with the eight highest DMI (1961, 1963, 1967, 1972, 1977, 1994, 1997, and 2006) are defined as the pIOD years and years with the eight lowest DMI (1960, 1964, 1974, 1975, 1996, 1998, 2005, and 2010) are defined as the nIOD years. The average DMI value for the pIOD is 1.27 °C, while that for the nIOD is − 0.88 °C. Their amplitudes are statistically different at the 95% confidence level. Note that Year 0 corresponds to the year that the IOD develops and Year 1 corresponds to the following year.

### ROMS

The model used in this study is the Regional Ocean Modeling System (ROMS)^38^ that covers the Indian Ocean (28°E–114°E, 46°S–32°N) with a horizontal resolution of 1/3° and 40 vertical sigma layers. For the eastern and southern boundary conditions, the mixed radiation-nudging boundary condition is applied^39^, and the monthly temperature, salinity, and horizontal velocity data from the Ocean Reanalysis System version 4 (ORAS4)^40^ are imposed. A turbulence closure scheme based on level 2.5 version of the turbulence closure model^41^ is adopted to calculate the vertical viscosity and diffusion coefficients as a function of turbulent kinetic energy, turbulent length scale, and a stability function. For the surface forcing, the model uses atmospheric variables from the Japanese 55-year Reanalysis Project (JRA55-do)^42^ and calculates the sensible and latent heat flux and evaporation using the Coupled Ocean–Atmosphere Response Experiment 3.0 algorithm^43^. The model is spun up for 30 years from the initial condition based on the World Ocean Atlas 2013 using climatological daily river runoff^44^, 3-hourly atmospheric forcing, and monthly lateral boundary forcing. Afterward, a hindcast run is conducted from 1958 to 2017 using interannually varying forcing. A more detailed description of the model is given by Kido et al.^45^.

The model can adequately reproduce the evolution of the observed IOD. For instance, the correlation coefficient of the simulated and observed DMI (Fig. S1) is very high at 0.91, although we note that this is not surprising considering the fact that the use of bulk formulae implicitly restores the simulated SST to the reanalysis data. However, it is important to note that the SST asymmetry in our model is not spuriously caused by the use of bulk formulae for surface heat fluxes. A scatterplot of net surface heat flux and SST anomalies over the eastern pole of the IOD for both the model and observation/reanalysis (ERSST and JRA-55) (Fig. S4) indicates that the model agrees with the observation/reanalysis in that the net surface heat flux tends to damp SST anomalies, i.e., surface heat flux anomalies are generally positive and warm the ocean when SST anomalies are negative, while surface heat flux anomalies tend to be negative for positive SST anomalies. Also, the model has a relatively good skill in simulating the seasonality of the IOD and surface and subsurface temperature/salinity anomalies associated with the pIOD^45^. Thus, the model reasonably reproduces the main features of the IOD including its asymmetry, and we may use this model for detailed investigation.

### Mixed layer heat budget

To understand the mechanism of the asymmetrical SST anomalies in the southeastern Indian Ocean, we conduct an online mixed layer heat budget analysis using the ROMS. The time evolution of the mixed layer temperature (MLT)^46^ can be written as follows:

1 $$\frac{\partial {T}_{M}}{\partial t}=-\frac{1}{h}{\int }_{-h}^{0}\left(u\frac{\partial T}{\partial x}\right)dz-\frac{1}{h}{\int }_{-h}^{0}\left(v\frac{\partial T}{\partial y}\right)dz-\frac{1}{h}{\int }_{-h}^{0}\left(w\frac{\partial T}{\partial z}\right)dz+\frac{1}{h}{\int }_{-h}^{0}{\nabla }_{h}\cdot \left({\kappa }_{h}{\nabla }_{h}T\right)dz -\frac{1}{h}{\left({\kappa }_{v}\frac{\partial T}{\partial z}\right)}_{z=-h}-\frac{\Delta T}{h}\frac{\partial h}{\partial t}+\frac{1}{\rho {C}_{p}h}\left({Q}_{net}-{Q}_{sw}\left(z=-h\right)\right) .$$

Here, ${T}_{M}$ is the MLT, and $h$ is the MLD, which is defined as a depth at which potential density is 0.01 kg/m^3^ greater than the sea surface density. We note that the MLD defined in the above way corresponds well with the turbocline depth; the annual mean turbocline depth and MLD are 58 m and 54 m, respectively, and their seasonal cycle is in phase (the minimum in March and the maximum in August) with the root mean square difference is 8 m. Here, the turbocline depth is defined as the depth at which the vertical diffusion coefficient falls below 0.005 m^2^ s^−1^, which is within the range of values used by previous studies^47,48^. Also, ${\kappa }_{h}$ and ${\kappa }_{v}$ are the horizontal and vertical diffusion coefficients, respectively, $\mathrm{\Delta T}$ is the temperature difference between the mixed layer and the entrained water, $\rho$ is the seawater density, and ${C}_{p}$ is the specific heat of the seawater. The net surface heat flux ${Q}_{net}$ includes the shortwave radiation, longwave radiation, sensible heat flux, and latent heat flux, and ${Q}_{sw}\left(z = - h\right)$ is the shortwave radiation at the bottom of the mixed layer. On the right-hand side, the first, second, and third terms are zonal, meridional, and vertical advection, respectively. The fourth and fifth terms represent horizontal and vertical mixing, respectively. The sixth term is the entrainment term, while the last term is the surface heat flux term. Except for the entrainment term, each term in Eq. (1) is calculated at every time step and accumulated as 3-day averaged value. The entrainment term is calculated by the method that guarantees the exact closure of the mixed layer heat budget^49^.

The horizontal advection term can be decomposed as follows:

2 $$\left(Horizontal \, advection\right)=-\left({U}^{^{\prime}}\frac{\partial \overline{{T }_{M}}}{\partial x}+{V}^{^{\prime}}\frac{\partial \overline{{T }_{M}}}{\partial y}\right)-\left(\overline{U}\frac{\partial {T }_{M}{^{\prime}}}{\partial x}+\overline{V}\frac{\partial {T }_{M}{^{\prime}}}{\partial y}\right) -\left({U}^{^{\prime}}\frac{\partial {T}_{M}{^{\prime}}}{\partial x}+{V}^{^{\prime}}\frac{\partial {T}_{M}{^{\prime}}}{\partial y}\right)+\left(Res.\right).$$

Here, $U$ and $V$ are monthly zonal and meridional current averaged over the mixed layer, respectively, an overbar represents the climatological mean, and a prime represents the anomaly. The first and second terms on the right-hand side are the linear advection terms, while the third term represents the nonlinear advection term. The last term is the difference between values estimated by an online calculation and offline diagnosis, arising from high-frequency variations of temperature and horizontal current fields and horizontal gradient of MLD.

To understand the physical processes responsible for vertical mixing term anomalies [i.e., the fifth term on the right-hand side of Eq. (1)], we have focused on three variables constituting this term: (1) MLD ($h$), (2) vertical diffusion coefficient at the bottom of the mixed layer ${(\left({\kappa }_{v}\right)}_{z=-h}$), and (3) differences between the MLT and temperature at the depth just below the mixed layer. Here, (3) is used to represent the vertical temperature gradient at the bottom of the mixed layer. Note that vertical diffusion coefficients are internally calculated in the model using the turbulence closure scheme, and are stored as 3-day averaged values, similar to other variables. Qualitatively, shoaling (deepening) of the mixed layer, increase (reduction) in vertical diffusion coefficients, and increase (reduction) in the vertical temperature differences lead to enhanced (suppressed) SST cooling by turbulent heat transport. For more quantitative discussions, it is necessary to conduct a decomposition that is similar to the horizontal advection term. Although the vertical mixing term is composed of three variables, a decomposition with three variables becomes too complicated and it is difficult to interpret. For this reason, we have decided to look at the following decomposition, because the difference in MLD anomalies between pIOD and nIOD is insignificant during the development phase:

3 $${-\left({\left({\kappa }_{v}\right)}_{z=-h}{T}_{diff}\right)}^{{^{\prime}}}=-{\left({\kappa }_{v}\right){^{\prime}}}_{z=-h}\overline{{T }_{diff}}-\overline{{\left({\kappa }_{v}\right)}_{z=-h}}{T}_{diff}{^{\prime}}-{\left({\kappa }_{v}\right)\mathrm{^{\prime}}}_{z=-h}{T}_{diff}{^{\prime}}$$

Here, ${T}_{diff}$ represents difference in temperature between the mixed layer and just below it.

### Skewness

The skewness is defined as

4 $$Skewness=\frac{{m}_{3}}{{\left({m}_{2}\right)}^{3/2}}$$

where ${m}_{k}$ is the *k*-th moment^11^.

### EQUINOO

To normalize heat budget terms, we use September–November EQUINOO, which is defined as the area averaged zonal wind stress anomalies over the equatorial Indian Ocean (60°E–90°E, 2.5°S–2.5°N)^50^.

## Supplementary Information

Supplementary Information.

## Data Availability

The ORAS4 is available from http://apdrc.soest.hawaii.edu/dods/public_data/Reanalysis_Data/ORAS4. The JRA55-do was downloaded from https://esgf-node.llnl.gov/projects/input4mips/. The source code and tools for input files of the ROMS are available from http://www.myroms.org/ and http://github.com/BobTorgerson/Pyroms. The ERSST data were obtained from the Asia–Pacific Data-Research Center of the International Pacific Research Center (http://apdrc.soest.hawaii.edu/data). The ROMS outputs are available from http://github.com/shokido/NKT2021.
