# Supplementary material for: Mechanisms of asymmetry in sea surface temperature anomalies associated with the Indian Ocean Dipole revealed by closed heat budget
Source: Sci Rep. 2021 Nov 25;11:22546. doi: 10.1038/s41598-021-01619-2 (PMC8617302; doi:10.1038/s41598-021-01619-2)
Supplement: Supplementary file 1 — Supplementary Information. [file 41598_2021_1619_MOESM1_ESM.pdf]

Supplementary information for

"Mechanisms of asymmetry in sea surface temperature anomalies associated with the Indian Ocean Dipole revealed by closed heat budget"

Mai Nakazato[1], Shoichiro Kido[2], and Tomoki Tozuka[1,2]

[1] Department of Earth and Planetary Science, Graduate School of Science, The University of Tokyo, Tokyo, Japan

[2] Application Laboratory (APL), Research Institute for Value-Added-Information Generation (VAiG), Japan Agency for Marine-Earth Science and Technology (JAMSTEC), Yokohama, Japan

Corresponding author address: Dr. Tomoki Tozuka, Department of Earth and Planetary Science, Graduate School of Science, The University of Tokyo, 7-3-1 Hongo, Bunkyo-ku, Tokyo 113-0033, Japan. E-mail: tozuka@eps.s.u-tokyo.ac.jp

Supplementary Figures

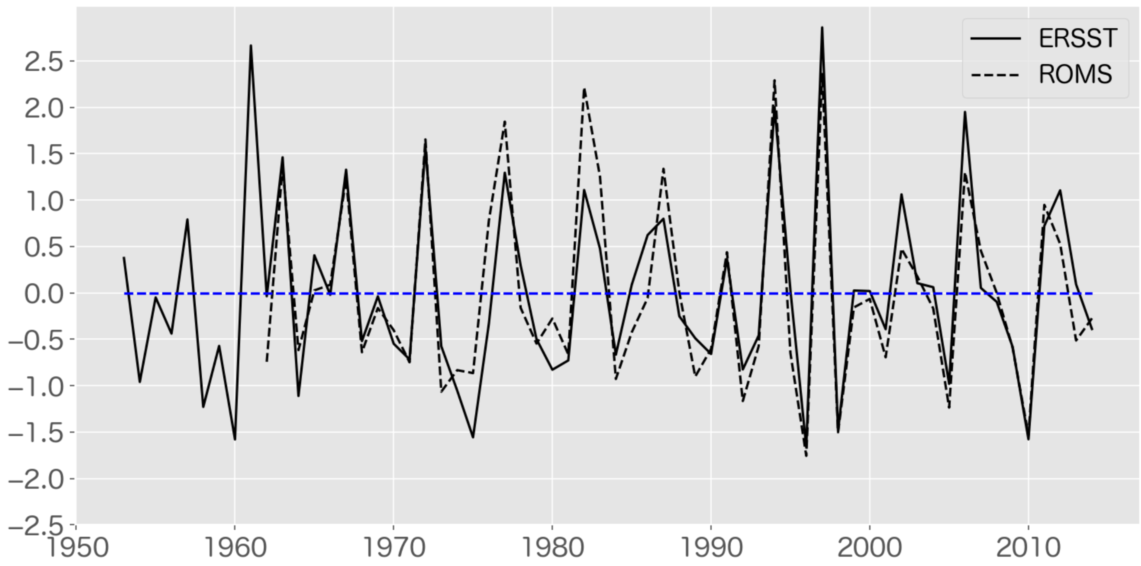

Figure S1: Time series of the normalized Dipole Mode Index (DMI) during September to November from the ERSST (solid line; from 1953 to 2014) and the ROMS (dashed line; from 1962 to 2014).

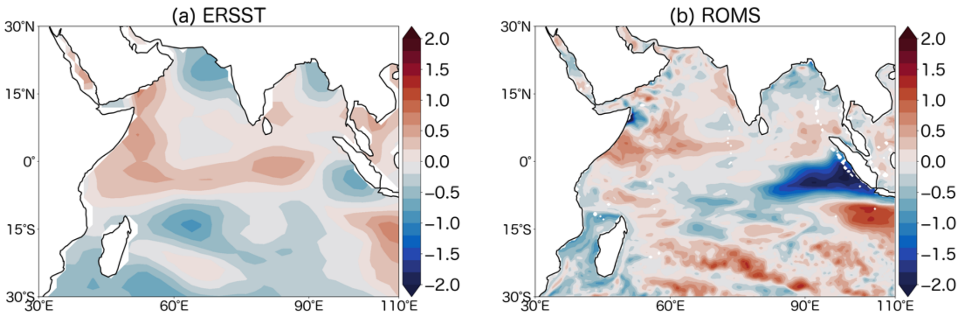

Figure S2: Skewness of sea surface temperature (SST) anomalies during September to November from (a) the ERSST and (b) the ROMS for 1959-2016. We have used Python 3.8.1 to prepare this figure.

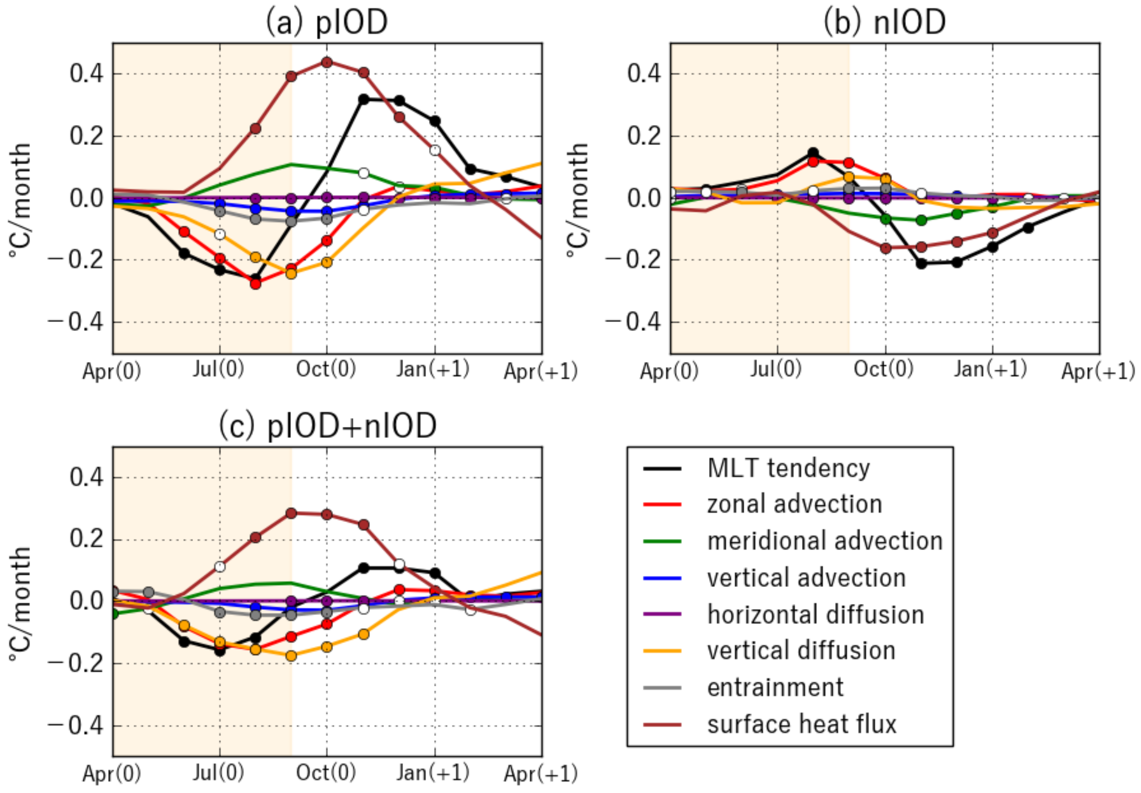

Figure S3: Time series of composited mixed layer temperature (MLT) tendency (black), zonal advection (red), meridional advection (green), vertical advection (blue), horizontal diffusion (purple), vertical diffusion (orange), entrainment (grey), and surface heat flux (brown) term anomalies (in °C/month) for (a) positive IOD (pIOD) and (b) negative IOD (nIOD) events. The sum of (a) and (b) is shown in (c). The closed (open) circles indicate anomalies significant at the 95% (90%) confidence level by a two-tailed t test. Year 0 represents the IOD year, while Year +1 signifies the following year. The development phase from April (0) to September (0) is shaded.

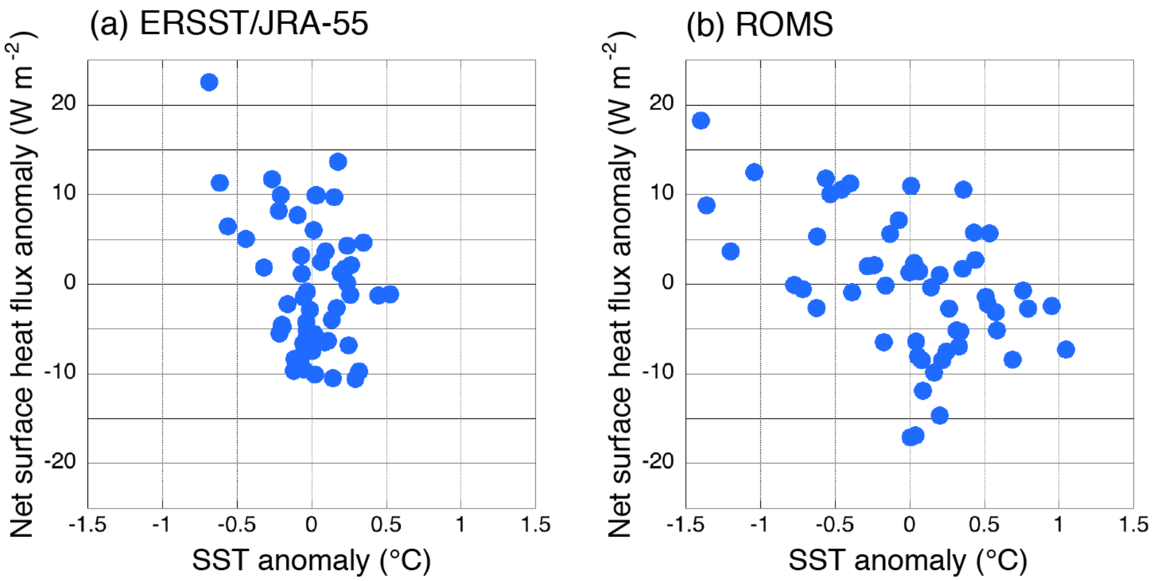

Figure S4: Scatterplot of net surface heat flux anomalies averaged over the development months (April-September) and SST anomalies during the peak season (September-November) over the eastern pole of the IOD for both (a) observation/reanalysis (ERSST and JRA-55) and (b) ROMS. Positive values in the net surface heat flux signify that the net surface heat flux warms the ocean.
